# Atezolizumab-bevacizumab rechallenge after immune checkpoint inhibitor-associated myocarditis in advanced hepatocellular carcinoma: a case report

**DOI:** 10.3389/fonc.2026.1931800

**Published:** 2026-09-10

**Authors:** Myriam Ayari, Sarra Ben Azouz, Meriem Ncir, Rim Bouyahia, Sarra Ben Rejeb, Mehdi Charfi, Taieb Jomni

**Affiliations:** 1 Gastroenterology Department, Internal Security Forces Hospital La Marsa, Tunis, Tunisia; 2 Radiology Department, Internal Security Forces Hospital La Marsa, Tunis, Tunisia; 3 Pathology Department, Internal Security Forces Hospital La Marsa, Tunis, Tunisia

**Keywords:** advanced hepatocellular carcinoma, atezolizumab-bevacizumab, hepatocellular carcinoma, ICI rechallenge, ICI-associated myocarditis, immune checkpoint inhibitor, immune-related adverse event (irAE)

## Abstract

**Background:**

atezolizumab–bevacizumab is the standard first-line systemic treatment for unresectable hepatocellular carcinoma (HCC). Immune checkpoint inhibitor (ICI)-associated myocarditis is a rare but potentially life-threatening immune-related adverse event. Evidence regarding ICI rechallenge after myocarditis remains limited, particularly in patients with HCC.

**Case presentation:**

We report the case of a 59-year-old man with hepatitis C–related cirrhosis and advanced HCC with portal vein tumor thrombosis, treated with atezolizumab-bevacizumab. After two treatment cycles, he developed non-fulminant ICI-associated myocarditis, characterized by chest pain, elevated cardiac biomarkers, and cardiac magnetic resonance imaging findings consistent with acute myocarditis. Atezolizumab was discontinued, and high-dose corticosteroid therapy was initiated, resulting in complete clinical, biochemical, and radiological recovery. The patient initially achieved a partial tumor response with normalization of alpha-fetoprotein (AFP). During temporary treatment interruption, he subsequently developed radiological disease progression, including enlargement of the hepatic lesion, portal vein tumor thrombosis, and extensive supra- and infradiaphragmatic necrotic lymphadenopathy. Following multidisciplinary evaluation and documented resolution of myocarditis, atezolizumab–bevacizumab rechallenge was undertaken under corticosteroid cover. The patient received six additional cycles with good tolerance, no recurrence of myocarditis, and stable disease on follow-up imaging.

**Conclusion:**

This case suggests that atezolizumab–bevacizumab rechallenge may be feasible in carefully selected patients with advanced HCC after recovery from non-fulminant ICI-associated myocarditis under close multidisciplinary supervision. It also highlights the importance of histological confirmation in atypical nodal progression during immunotherapy, particularly when imaging findings may be confounded by alternative explanations.

## Introduction

Atezolizumab in combination with bevacizumab has become a standard first-line systemic therapy for unresectable hepatocellular carcinoma (HCC), demonstrating improved overall survival and progression-free survival compared with sorafenib (1, 2). This therapeutic advancement has significantly expanded the use of immune checkpoint inhibitors (ICI) in patients with underlying chronic liver disease and cirrhosis, a population that may present increased clinical complexity and vulnerability to treatment-related toxicity. However, ICIs are associated with a broad spectrum of immune-related adverse events, among which myocarditis is rare but potentially life-threatening. Current management relies on early recognition, prompt discontinuation of immunotherapy, and initiation of high-dose corticosteroid therapy (3–5). Despite increasing clinical experience with ICI-related toxicities, myocarditis remains one of the most feared complications due to its abrupt onset, potential severity, and limited evidence guiding subsequent oncologic management. Evidence regarding the safety and feasibility of ICI rechallenge following resolution of myocarditis remains scarce and is primarily limited to case reports and small series.

In parallel, extrahepatic lymph node metastases from HCC are uncommon, particularly when involving extensive supra- and infradiaphragmatic nodal stations. Such atypical metastatic patterns may mimic alternative malignancies or inflammatory conditions, thereby complicating staging accuracy and therapeutic decision-making. In these contexts, histological confirmation can be essential in selected cases to guide treatment strategy and avoid misclassification (6). Furthermore, the coexistence of systemic immune toxicity and suspected disease progression raises complex therapeutic dilemmas, particularly regarding continuation versus interruption of effective immunotherapy.

We report the case of a 59-year-old man with hepatitis C–related cirrhosis and advanced HCC who developed non-fulminant atezolizumab-associated myocarditis. During treatment interruption, he experienced biopsy-confirmed nodal disease progression. Following multidisciplinary discussion and documented complete cardiac recovery, a rechallenge with atezolizumab–bevacizumab was successfully undertaken without recurrence of myocarditis. This case highlights the potential feasibility of carefully selected ICI rechallenge after immune-mediated myocarditis, as well as the importance of multidisciplinary decision-making in balancing oncologic control and cardiac safety.

## Case presentation

**Figure 1:**
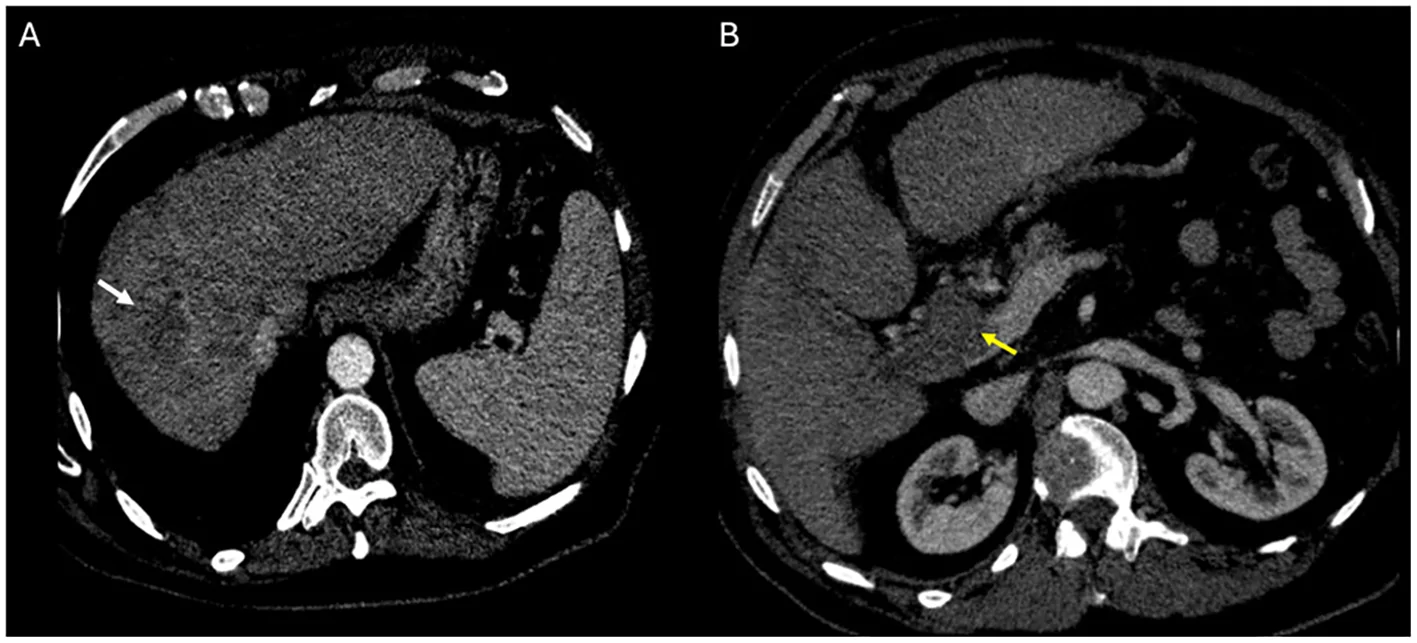
Baseline contrast-enhanced CT (February 2024). **(A)** Dominant hepatocellular carcinoma involving hepatic segments VII–VIII (white arrow). **(B)** Extensive portal vein tumor thrombosis (yellow arrow).

A 59-year-old man with compensated hepatitis C–related cirrhosis and type 2 diabetes mellitus treated with metformin was diagnosed with advanced hepatocellular carcinoma in February 2024. Initial imaging revealed a dominant hepatic mass involving segments VII and VIII associated with extensive portal vein tumor thrombosis (**Figure 1**). Serum alpha-fetoprotein (AFP) was markedly elevated at 6,536 ng/mL. Liver biopsy confirmed well- to moderately differentiated HCC arising in a cirrhotic liver. No extrahepatic disease was initially documented at baseline staging.

First-line systemic treatment with atezolizumab (1,200 mg) plus bevacizumab (15 mg/kg) every three weeks was initiated. Baseline cardiovascular assessment, including electrocardiography, transthoracic echocardiography, serum troponine I hypersensible (hs-cTnI), and NT-proBNP levels, was unremarkable. Given the known risk of immune-related cardiotoxicity, baseline evaluation was considered essential prior to ICI initiation. The patient received two cycles of treatment in May 2024.

**Figure 2:**
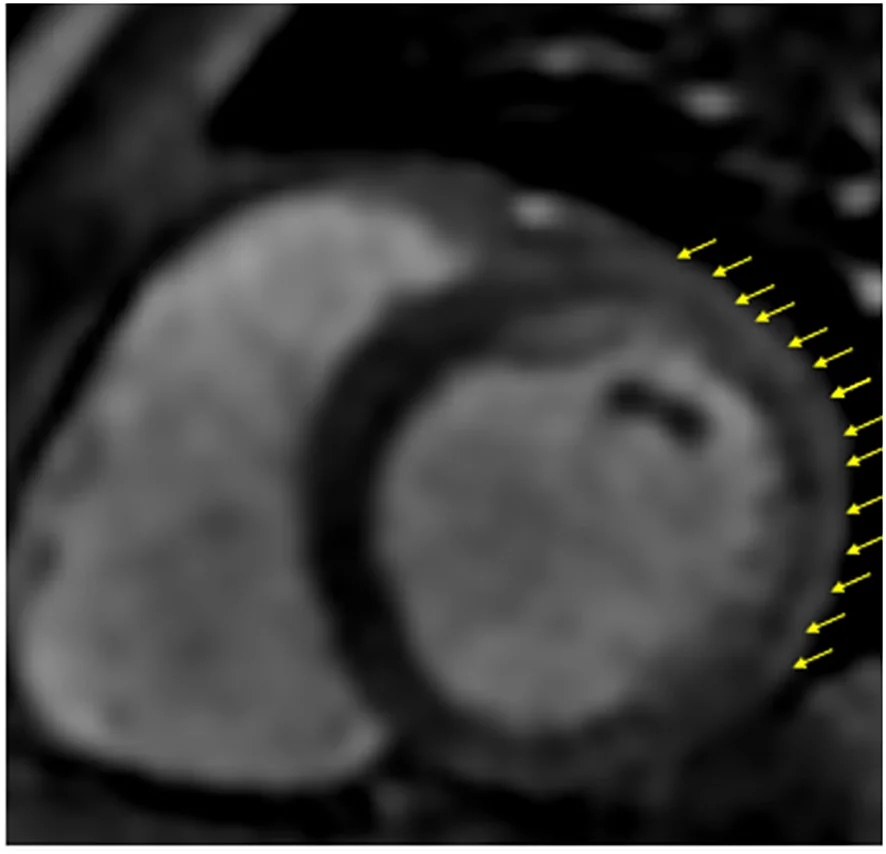
Cardiac MRI performed two weeks after the second cycle of atezolizumab-bevacizumab (June 2024) showing subepicardial late gadolinium enhancement (arrows) involving the lateral wall of the left ventricle, consistent with acute immune checkpoint inhibitor-associated myocarditis.

Two weeks after the second cycle, the patient presented with precordial chest pain and fatigue. Laboratory investigations demonstrated creatine phosphokinase elevation to approximately eight times the upper limit of normal and a troponin (hs-cTnI) level of 175 ng/L (upper limit of normal <14 ng/L). Concomitant ICI-related myositis was considered given the presence of myalgias, muscle weakness, and an approximately eight-fold elevation of CPK. Other potential causes of CPK elevation, including intense physical exertion, trauma, infection, alcohol consumption, and newly introduced medications, were clinically excluded. Thyroid function tests were normal. Electrocardiography showed no abnormalities, and transthoracic echocardiography remained normal. Cardiac magnetic resonance imaging (MRI) revealed linear patchy late gadolinium enhancement involving the lateral wall of the left ventricle, consistent with myocarditis (**Figure 2**). The left ventricle was mildly dilated, with a slightly reduced ejection fraction of 52%, without evidence of ischemic injury. Based on ESMO severity criteria, the event was classified as grade 2 immune checkpoint inhibitor-associated myocarditis. The diagnosis was consistent with a subacute, non-fulminant immune-mediated myocarditis.

Following cardio-oncology consultation, atezolizumab was discontinued. Corticosteroid therapy and an angiotensin-converting enzyme inhibitor were initiated for immune checkpoint inhibitor-related myocarditis. The patient was treated with intravenous methylprednisolone (1 g/day) for three consecutive days, followed by oral prednisone at 1 mg/kg/day (60 mg/day). This dose was maintained for one month and then gradually tapered by 5 to 10 mg per week until complete discontinuation after three months with favorable clinical evolution. Follow-up cardiac MRI demonstrated complete resolution of the previously observed subepicardial enhancement, supporting radiological recovery from active myocarditis. Serial measurements demonstrated progressive normalization of hs-cTnI to 9.7 ng/L by August 2024, accompanied by normalization of CPK to 213 U/L following corticosteroid therapy. The parallel improvement of both biomarkers further supported the possibility of concomitant immune-mediated muscle involvement. During this period, one additional cycle of bevacizumab monotherapy was administered without complications.

From an oncological perspective, the patient achieved a favorable treatment response, with normalization of serum alpha-fetoprotein (AFP) levels and a partial radiological response according to RECIST version 1.1. The dominant hepatic lesion decreased in size from 60 × 45 mm to 40 × 35 mm. Considering the favorable tumor response and the recent immune checkpoint inhibitor-associated cardiac toxicity, surveillance was considered the most appropriate management strategy.

**Figure 3:**
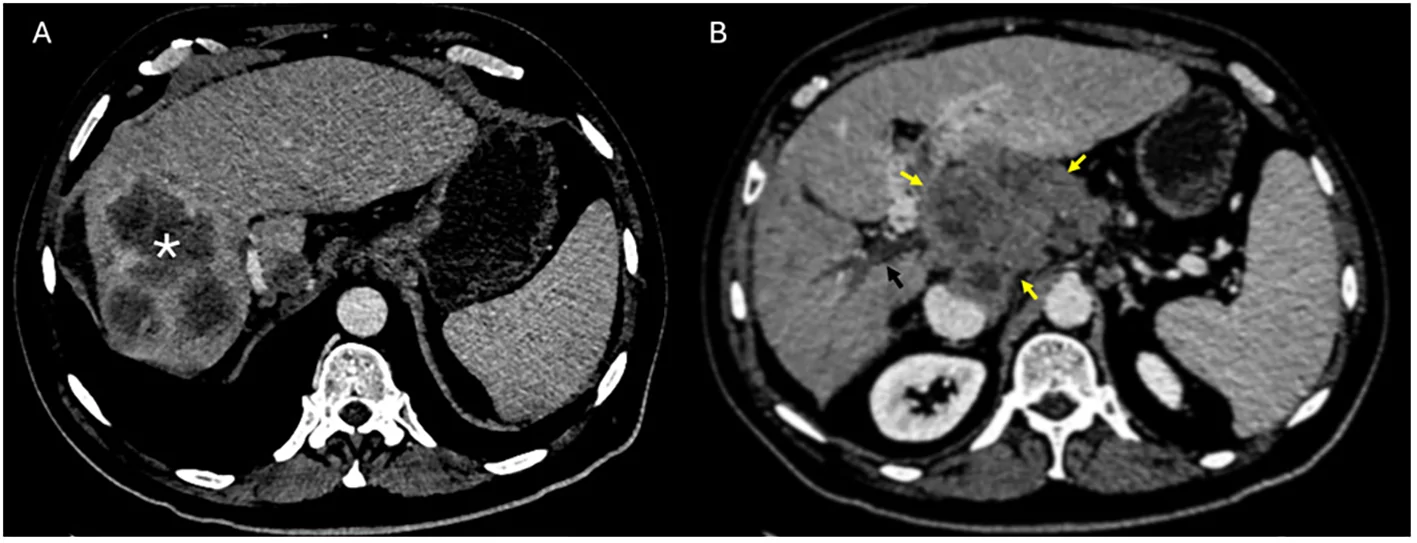
Follow-up CT scan (May 2025). **(A)** Tumor progression with increased size of hepatic lesions (white asterisk). **(B)** Necrotic hilar lymphadenopathy (yellow arrows) and extension of portal vein tumor thrombosis (black arrow).

**Figure 4:**
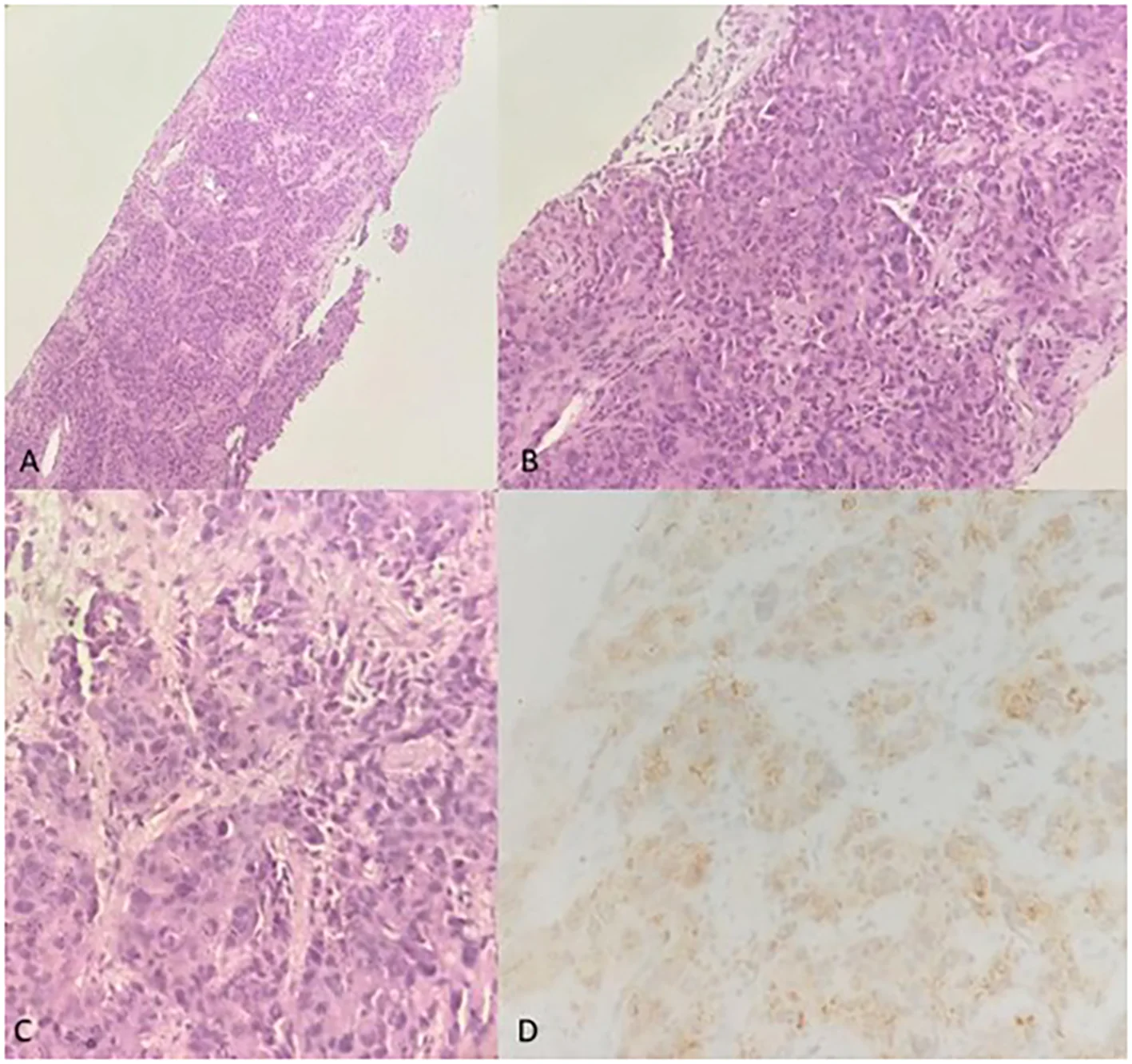
Histopathological examination of subsequent lymph-node biopsy confirming metastatic poorly differentiated hepatocellular carcinoma. **(A)** Solid carcinomatous proliferation (HE×10). **(B)** Tumor nests and sheets (HE×20). **(C)** Large polygonal cells with eosinophilic cytoplasm, nuclear pleomorphism, and mitoses (HE×40). **(D)** Tumor cells showing weak diffuse granular cytoplasmic expression for Hepar-1 (IHC×40).

During follow-up, the patient developed right upper quadrant pain. Despite persistently normal AFP levels, computed tomography revealed disease progression characterized by enlargement of the hepatic lesion, extension of portal vein tumor thrombosis, and extensive supra- and infradiaphragmatic necrotic lymphadenopathy forming a large nodal conglomerate (**Figure 3**). The discordance between AFP levels and radiological progression underscored the importance of imaging-based surveillance in advanced HCC. Because such extensive nodal involvement is uncommon in HCC and raised alternative diagnostic considerations, biopsy of the lymph node mass was performed and confirmed metastatic HCC (**Figure 4**).

Given the preserved liver function (Child–Pugh A5), the limited systemic treatment options available, lack of superior therapeutic alternatives, previous marked clinical benefit from atezolizumab-bevacizumab, and documented radiological resolution of myocarditis, reintroduction of immunotherapy was discussed during a multidisciplinary meeting involving hepatologists, oncologists, and cardiologists. The decision-making process carefully balanced the high oncologic benefit previously observed against the uncertain but potentially severe risk of myocarditis recurrence. After a detailed discussion regarding the potential risk of recurrent myocarditis, the patient provided informed consent for immune checkpoint inhibitor rechallenge.

Prior to immunotherapy rechallenge in May 2025, a comprehensive cardiac evaluation was performed, including clinical assessment, electrocardiography, cardiac biomarkers (troponin and NT-proBNP), and transthoracic echocardiography, which demonstrated normal cardiac function. A repeat cardiac MRI was not performed because the patient had achieved complete clinical recovery, normalization of cardiac biomarkers, and documented complete resolution of myocarditis on follow-up cardiac MRI.

To reduce the risk of myocarditis recurrence, the patient received a prophylactic single oral dose of prednisone (40 mg) before each immune checkpoint inhibitor infusion. Given the history of grade 2 ICI-associated myocarditis, a strict multidisciplinary cardiac monitoring protocol was implemented throughout the rechallenge period. Before each cycle of atezolizumab-bevacizumab, a systematic clinical assessment was performed to screen for potential cardiac symptoms, including chest pain, dyspnea, palpitations, syncope, and signs of heart failure. Electrocardiography and serial measurement of cardiac biomarkers, including hs-cTnI, NT-proBNP, and CPK, were performed before each treatment cycle to detect early evidence of recurrent myocardial or muscle injury. Transthoracic echocardiography was repeated during follow-up according to the clinical and biological findings. The monitoring strategy was determined based on the patient’s previous ICI-associated myocarditis and the potential risk of recurrence during ICI rechallenge, with particular emphasis on early detection of cardiac toxicity. Any new cardiac symptoms or significant increase in cardiac biomarkers would have prompted treatment interruption and urgent cardiological reassessment.

The patient subsequently completed six additional cycles of atezolizumab-bevacizumab from May to December 2025 without recurrence of myocarditis or other significant immune-related adverse events. Follow-up imaging showed stable disease, suggesting sustained oncological benefit despite previous immune-related toxicity. All cardiac assessments remained normal throughout treatment, with persistently negative cardiac biomarkers and no electrocardiographic or echocardiographic abnormalities suggestive of recurrent myocarditis. The overall clinical course is summarized in **Table 1**.

**Table 1:** Clinical timeline of the patient’s clinical course.

Time from atezolizumab–bevacizumab initiation	Clinical event
Day 0 (May 8, 2024)	Initiation of first-line atezolizumab–bevacizumab (cycle 1).
Day 21 (May 29, 2024)	Cycle 2 of atezolizumab–bevacizumab. Normalization of AFP.
Day ~42 (June 2024)	Grade 2 ICI-associated myocarditis diagnosed based on chest pain, elevated cardiac biomarkers, and cardiac MRI findings. Atezolizumab discontinued. High-dose intravenous methylprednisolone initiated.
Month 3 (August 2024)	Complete clinical recovery with normalization of cardiac biomarkers and resolution of myocarditis on follow-up cardiac MRI. Bevacizumab continued as monotherapy. Partial radiological tumor response.
Month 4 (September 2024)	Corticosteroid taper completed. Surveillance indicated
Month 12 (May 2025)	Radiological disease progression. Lymph-node biopsy confirmed metastatic HCC. Atezolizumab–bevacizumab rechallenge supported by multidisciplinary discussion after complete cardiac recovery.
Months 12-19 (May–December 2025)	Six additional cycles of atezolizumab–bevacizumab administered without recurrence of myocarditis. Stable disease maintained with normal cardiac monitoring.

AFP, alpha-fetoprotein; HCC, hepatocellular carcinoma; ICI, immune checkpoint inhibitor; MRI, magnetic resonance imaging.

## Discussion

Immune checkpoint inhibitor (ICI)-associated myocarditis is a rare but potentially severe immune-related adverse event, typically occurring early after treatment initiation, most often within the first two cycles. Current recommendations from the European Society for Medical Oncology (ESMO), the American Society of Clinical Oncology (ASCO), and cardio-oncology expert groups advocate immediate treatment interruption and prompt initiation of high-dose corticosteroids, with escalation to additional immunosuppressive therapies in refractory cases (3, 7, 8). However, while acute management is well defined, the optimal oncologic strategy after recovery remains uncertain, particularly in patients who continue to require effective systemic therapy. Evidence regarding ICI rechallenge after myocarditis is limited to case reports, small case series, and retrospective analyses (5, 9, 10).

A review of the published literature identified 16 patients who underwent ICI rechallenge after myocarditis across nine reports. Recurrence of myocarditis or cardiac symptoms has been described in a subset of cases, suggesting that rechallenge may be feasible but carries a non-negligible risk (9). More recent prospective data provide further support for this approach. In a prospective cohort of 138 patients with ICI-associated myocarditis, 14 underwent ICI rechallenge after cardiac recovery, with only one recurrence of grade 3 myocarditis and no severe heart failure reported (11). These findings suggest that rechallenge may be feasible in carefully selected patients, although the small number of rechallenged patients limits the generalizability of the results. Rechallenge decisions should therefore remain individualized and based on multidisciplinary assessment, considering initial myocarditis severity, completeness of clinical and biochemical recovery, and the availability of effective alternative therapies (5, 10, 12, 13). Successful rechallenge has been reported after both moderate and fulminant myocarditis across different tumor types, including melanoma and mismatch repair-deficient gastrointestinal malignancies. However, substantial heterogeneity exists regarding the type of ICI, initial toxicity severity, and monitoring strategies during re-exposure, limiting generalizability (5, 10, 12, 13).

In the present case, several favorable factors supported rechallenge, including the non-fulminant presentation of myocarditis, complete clinical recovery, normalization of cardiac biomarkers, and radiological resolution on cardiac MRI. Additional considerations included preserved hepatic function (Child–Pugh A5), lack of superior systemic alternatives, and the previously marked oncologic response to atezolizumab–bevacizumab. Following multidisciplinary discussion, rechallenge was undertaken and was well tolerated, with no recurrence of cardiac toxicity during six subsequent cycles. The role of corticosteroid prophylaxis during rechallenge remains uncertain. Current guidelines do not recommend steroid prophylaxis as a standard approach when restarting ICIs after myocarditis (3, 7, 8). Reported practices are highly heterogeneous, ranging from complete steroid discontinuation before rechallenging to low-dose maintenance or prolonged tapering strategies (9, 10, 12). Therefore, the prophylactic corticosteroid regimen used in this case should be interpreted as an individualized precautionary strategy rather than a guideline-supported standard.

Although myocarditis associated with atezolizumab–bevacizumab has been reported in HCC, prior publications have primarily focused on acute diagnosis and management rather than immunotherapy reintroduction (4). More recently, retrospective data suggest that atezolizumab–bevacizumab rechallenge may be feasible in selected HCC patients after prior treatment discontinuation, although immune-related myocarditis was not specifically represented among the reported toxicities (14). To our knowledge, this case adds to the existing literature by describing successful rechallenge with the atezolizumab–bevacizumab combination after confirmed ICI-associated myocarditis in advanced HCC, with no recurrence of cardiac toxicity during six subsequent cycles. It further contributes to the emerging evidence supporting carefully selected rechallenge strategies following immune-related cardiac toxicity.

Another important aspect of this case is the pattern of disease progression observed during treatment interruption. Extrahepatic spread of HCC most commonly involves the lungs, lymph nodes, bone, and adrenal glands. Although lymph node metastases are recognized, extensive supra- and infradiaphragmatic nodal involvement remains uncommon and may raise alternative diagnostic considerations (15). In large series, lymph node metastases are reported in a minority of patients, and distant nodal dissemination is associated with poor prognosis (16). In this context, histological confirmation is particularly important when imaging findings are atypical and when alternative diagnoses such as second primary malignancy, inflammatory disease, or treatment-related phenomena are possible (6, 17, 18).

In patients receiving immunotherapy, pseudoprogression represents an additional diagnostic challenge. This phenomenon has been described in HCC treated with ICIs, including atezolizumab–bevacizumab, and may manifest as transient radiological worsening before tumor regression (19, 20). Pathological correlates, including immune cell infiltration, have been reported in some cases, supporting an immune-mediated mechanism of apparent progression (21). These observations underscore that radiological progression during or after immunotherapy should not be interpreted in isolation. In our patient, true disease progression was supported by the overall clinical and radiological course, with enlargement of the hepatic lesion, extension of portal vein tumor thrombosis, and extensive supra- and infradiaphragmatic lymphadenopathy after an initial partial response. Lymph node biopsy confirmed metastatic HCC, further supporting true progression during treatment interruption rather than pseudoprogression. This distinction was critical for therapeutic decision-making and for the subsequent risk–benefit assessment supporting rechallenge.

Overall, this case highlights two clinically relevant messages. First, atezolizumab–bevacizumab rechallenge may be feasible after complete recovery from non-fulminant ICI-associated myocarditis in carefully selected patients, provided that multidisciplinary evaluation and close cardiac monitoring are ensured. Second, atypical nodal progression during immunotherapy warrants histological confirmation, particularly when pseudoprogression remains a plausible alternative explanation.

This report has several limitations. First, it describes the experience of a single patient, limiting the generalizability of the findings. Second, although the diagnosis of myocarditis was supported by clinical presentation, serial cardiac biomarkers, and cardiac MRI findings, endomyocardial biopsy was not performed. Third, the contribution of the prophylactic single-dose corticosteroid regimen to the favorable outcome remains uncertain and cannot be distinguished from the patient’s complete recovery before rechallenge. Finally, the duration of follow-up after rechallenge was limited, precluding conclusions regarding the long-term safety and durability of this strategy. Therefore, rechallenge after ICI-associated myocarditis should remain an individualized multidisciplinary decision until larger prospective studies become available.

In conclusion, this case describes atezolizumab–bevacizumab rechallenge in a patient with advanced hepatocellular carcinoma following complete recovery from non-fulminant immune checkpoint inhibitor-associated myocarditis. During the reported follow-up, rechallenge was completed without recurrence of clinically recognized myocarditis. This case also underscores the importance of integrating oncologic, cardiologic, and pathological data into complex therapeutic decision-making during immunotherapy. In addition, it highlights the value of histological confirmation in atypical nodal progression patterns, where both true metastatic disease and immune-related imaging phenomena such as pseudoprogression should be considered. Further studies are required to evaluate the safety, efficacy, patient selection, and monitoring strategies for ICI rechallenge after myocarditis.

## Patient perspective

The patient was fully informed about the diagnosis, the potential risks and benefits of immunotherapy rechallenge, and the planned cardiac monitoring strategy. He was actively involved in the therapeutic decision-making process and participated in the multidisciplinary discussions regarding treatment options. After careful consideration, he agreed to resume immunotherapy.

## Data Availability

The original contributions presented in the study are included in the article/**Supplementary Material**. Further inquiries can be directed to the corresponding author.
